# Supplementary material for: Characterization of social frailty domains and related adverse health outcomes in the Asia-Pacific: a systematic literature review
Source: PeerJ. 2024 Mar 15;12:e17058. doi: 10.7717/peerj.17058 (PMC10946386; doi:10.7717/peerj.17058)
Supplement: Supplemental Information 1 [file peerj-12-17058-s001.docx]

**Supplementary Documents 1:** PRISMA Checklist

| **Section and Topic** | **Item #** | **Checklist item** | **Location where item is reported (Page/Line No)** |
| --- | --- | --- | --- |
| **TITLE** | | |  |
| Title | 1 | Characterization of Social Frailty Domains and Related Adverse Health Outcomes: A Systematic Literature Review. | Title: Page 1; Line 1 |
| **ABSTRACT** | | |  |
| Abstract | 2 | Frailty is a significant healthcare challenge worldwide, increasing interest in developing more assessment tools covering distinct domains of frailty. Recently, there has been a growing awareness of a correlation between social variables and frailty in older people. However, there is a lack of understanding of the social domains of frailty and the related adverse outcomes, particularly in the Asia-Pacific settings. The current study aimed to characterize the social frailty domains and their health outcomes by overviewing the frailty screening tools in older people in Asia-Pacific communities. A systematic review, using the PRISMA guideline, was conducted on papers published between 2002 and 2023 from three electronic databases, including PubMed, Scopus, and ScienceDirect, using MeSH terms and the inclusion and exclusion criteria. A manual search was conducted for the references of the complete full-text content using Google Scholar. A total of 31 studies were included in the thematic analysis, from which 16 screening tools measuring six social domains were reviewed. The most represented social domains in the tools reviewed were social networks, followed by social activities, social support, financial difficulties, social roles, and socioeconomic, arranged in four categories: social resources, social needs, social behaviors (or social activities), and general resources. The six social domains predicted mortality, physical difficulties, and disability incidence. Other adverse health outcomes were also associated with these social domains, including cognitive disorder (n= 5 domains), mental illness (n= 5 domains), nutritional disorders (n= 5 domains), dementia (n= 4 domains), oral frailty (n= 3 domains), hearing loss (n= 3 domains), obesity (n= 3 domains), and chronic pain (n= 3 domains). Overall, social frailty is a complex construct with multiple dimensions, including the frailty of social and general resources, social behaviors, and social needs, leading to several physical, mental, and cognitive health disorders. The findings contribute to understanding the conceptual framework of social frailty syndrome in older people and its related health outcomes. The significance of this review lies in linking gerontology to social health and health sciences. | Abstract; Page 1; Lines 4-31 |
| **INTRODUCTION** | | |  |
| Rationale | 3 | There is an increased number of SLRs investigating the prevalence of frailty, its assessment tools, and its various health outcomes. Despite this truth, there is a significant knowledge gap regarding the social dimensions of frailty and the possible health outcomes related to each domain, especially in the Asia-Pacific context. Besides, there is a lack of studies reviewing the existing social frailty assessment tools, which critically contribute to understanding social frailty and its domains, especially in the Asia-Pacific region. Besides, there is a lack of understanding of the social domains of frailty and the related adverse outcomes, particularly in the Asia-Pacific settings. | Introduction: Rationale of the systematic review; Page 4; Lines 109-132. |
| Objectives | 4 | The current study aimed to characterize the social frailty domains and their health outcomes by overviewing the frailty screening tools in older people in Asia-Pacific communities. In line with the objective the following research questions were addressed: (1) What are the existing screening tools for assessing social frailty syndrome in older people living in Asia-Pacific communities? (2) What are the main domains included in these social frailty screening tools? (3) What are the adverse health outcomes related to the social frailty domains in older people in Asia-Pacific communities? | Introduction: Page 3; Lines 90-93.  &  Literature Survey Methodology; Page 5; Lines 145-149. |
| **METHODS** | | |  |
| Eligibility criteria | 5 | The study’s inclusion criteria were:  (a) Publication year and language of the study: studies published between 2002 and 2023 in the English language.  (b) Study type: articles published in peer-reviewed journals (e.g., quantitative, qualitative, or mixed-methods).  (c) Location: studies’ location must be in the Asia-Pacific region.  (d) Study scope: selected studies must include the assessment of social domain of frailty.  (e) Target group: sample of the studies must be community-dwelling older adults aged 60 years or older. | Literature Survey Methodology: Inclusion and exclusion criteria; Page 6; Lines 177-190. |
| Information sources | 6 | Three electronic databases were reviewed:  (1) PubMed  (2) Scopus  (3) ScienceDirect  A manual search was conducted for the references of the complete full-text content using Google Scholar. | Literature Survey Methodology: Search strategy; Page 5; Lines 161-164. |
| Search strategy | 7 | The following search string and MeSH terms were used:  (“social frailty” OR “frailty social domain” OR “frailty social factor” OR “social vulnerability” OR “social vulnerabilities”) AND (“frailty screening tools” OR “frailty assessment tools” OR instruments OR “assessment tools” OR “screening tools”) AND (“risk factors” OR negative impacts OR “adverse outcomes” OR “adverse health outcomes”) AND (“Aged”[MeSH Terms] OR “older adults”[MeSH Terms] OR elderly[MeSH Terms]) AND (Asia-Pacific[MeSH Terms] OR “Southeastern Asia communit*”[MeSH Terms]).  Truncation, Boolean operators, parentheses, wildcards, quotation marks, and MeSH terms or search terms related to the described keywords were applied whenever possible.  The search string was used only for the title, abstract, and keywords. | Literature Survey Methodology: Search strategy; Page 5; Lines 164-174. |
| Selection process | 8 | In PRISMA's first and second steps (identification and screening), two independent reviewers reviewed the papers' titles and abstracts to include relevant articles. Studies written in English between 2002 and 2023 were included, and duplicates were excluded. In the title and abstract screening, studies were included if they described and had the social frailty determinants, assessment tools, and domains or if they included a combination of these variables. Then, all articles selected by either one reviewer or both of them were included in the next step.  In the second step of PRISMA (eligibility), the included papers from the previous step were retrieved in full text from the same independent reviewers using the eligibility criteria mentioned above. A manual search was conducted on the reference lists of the selected articles to avoid any missing articles in the search process during the previous step. Ultimately, the two independent reviewers engaged in a comprehensive discussion regarding the papers and reached a consensus on their inclusion. If an agreement could not be achieved, the ultimate determination was made by a third impartial reviewer. After selecting the included studies, a summary table was reported to present data from the studies, as shown in Table 3. The included studies were then analyzed and synthesized by the two authors using Atlas.ti.9. All five authors had approved the steps of the search protocol. | Literature Survey Methodology: Study selection; Pages 6 & 7; Lines 193-210. |
| Data collection process | 9 | The two reviewers extracted all data from the selected papers that could be related to social frailty, its domains, health outcomes, and assessment tools. To include a broad scope of factors related to social frailty, the data extracted is based on the definition and conceptual framework of social frailty. For example, each criterion included in the existing frailty index and showed an effect on social frailty was reviewed, such as socioeconomic status, loneliness, or isolation. Gobbens's and Bunt's frameworks were used to synthesize the criteria found and based on the factors highlighted in the conceptual framework of the current study. This conceptual background was used to have a comprehensive framework that integrates all domains and assessment tools associated with social frailty. Therefore, three themes to extract the data were (a) frailty assessment tools, (b) social domains of frailty, and (c) adverse outcomes related to social frailty and its domains. The three themes were identified based on the systematic review questions and objectives. For the first theme (frailty assessment tools), the data was synthesized and analyzed in sixteen categories describing each assessment tool's main definition and social frailty domains. The data was synthesized in six codes and four categories in the second theme (social domains of frailty). The third theme (adverse outcomes related to social frailty and its domains) included eleven codes (health outcome) and four categories. The four categories of the second and third themes included (1) social resources, (2) social needs, (3) social behaviors (or social activities), and (4) general resources. | Literature Survey Methodology: Data extraction and synthesis; Page 9; Lines 213-230. |
| Data items | 10 | Three themes to extract the data were (a) frailty assessment tools, (b) social domains of frailty, and (c) adverse outcomes related to social frailty and its domains. The three themes were identified based on the systematic review questions and objectives. For the first theme (frailty assessment tools), the data was synthesized and analyzed in sixteen categories describing each assessment tool's main definition and social frailty domains. The data was synthesized in six codes and four categories in the second theme (social domains of frailty). The third theme (adverse outcomes related to social frailty and its domains) included eleven codes (health outcome) and four categories. The four categories of the second and third themes included (1) social resources, (2) social needs, (3) social behaviors (or social activities), and (4) general resources. | Literature Survey Methodology: Data extraction and synthesis; Page 9; Lines 223-230. |
| Study risk of bias assessment | 11 | Two reviewers independently carried out a quality assessment of the included studies using the Newcastle-Ottawa Scale (NOS). Any disagreements regarding the included studies were resolved in an online meeting among the authors. The NOS is a risk-of-bias tool for nonrandomized studies, which assesses three domains, including study selection (four factors), study comparability (one factor), and exposure and outcomes (two factors). In general, studies with a low risk of bias (NOS score from 6 to 7 points) were included in the current review. Studies with a high risk of bias (NOS score from 4 to 5 points) and a very high risk of bias (NOS score from 0 to 3 points) were excluded, see Supplementary Document 2. | Literature Survey Methodology: Study quality assessment; Pages 7 & 8; Lines 233-240. |
| Synthesis methods | 13a | All studies were reviewed based on the mentioned three themes using thematic, tabular, and figural analysis via Atlas.ti.9. | Literature Survey Methodology: Data extraction and synthesis; Pages 7. |
|  | 13b | All studies were analyzed based on the mentioned three themes using thematic, tabular, and figural analysis via Atlas.ti.9. |  |
|  | 13c | Atlas.ti.9 and excel were used for the statistical data in each theme. |  |
|  | 13d | Each study result was analyzed individually to extract the relevant data (meta-analysis) |  |
| Reporting bias assessment | 14 | Specify any assessment of risk of bias that may affect the cumulative evidence. | Literature Survey Methodology: Study quality assessment; Pages 7. |
| Certainty assessment | 15 | Two reviewers independently carried out a quality assessment of the included studies using the Newcastle-Ottawa Scale (NOS), as mentioned above. |  |
| **RESULTS** | | |  |
| Study selection | 16a | In the first step, a total of 1,896 text materials were initially identified through the search using the mentioned databases. Besides, through manual search, seven additional materials were included from the available reference lists of the final selected text materials. After eliminating duplicate studies and including journal articles published in English between 2002 and 2023, 942 studies remained; however, 820 were excluded based on title and abstract. In the second step, a full text of 122 studies was read by the two reviewers, leading to the inclusion of 31 studies and the exclusion of 91 studies, as they included frailty in countries other than the Asia-Pacific countries (n= 29), involved samples of less than 60 years of age (n=3), involved samples of hospitalized or patient older people (n= 20), did not contain relevant findings in the social domain of frailty (n= 18), were review studies (n= 18), and failed in quality appraisal (NOS total quality score was less than 6; n= 3). The rejected studies mostly could not provide information on outcomes, ascertainment of exposure, controlled confounding factors, or sample size. | Results; Page 8; Lines 244-257. |
|  | 16b | Only three studies excluded due to failed in quality appraisal (NOS total quality score was less than 6). The rejected studies mostly could not provide information on outcomes, ascertainment of exposure, controlled confounding factors, or sample size. | Results; Page 8; Lines 244-257. |
| Study characteristics | 17 | For the individual studies, 11 were cross-sectional studies (secondary data analysis), 13 were longitudinal studies (secondary data analysis), 6 were cross-sectional surveys (primary data analysis), and 1 was the experts’ interview method (preliminary data analysis; see Appendix 1). These studies were conducted in five Asia-Pacific countries, including Japan (n=17), China (n=6), South Korea (n=4), Singapore (n=2), and Thailand (n=1). Besides that, one of the selected studies was conducted in a general Asia-Pacific community of older adults without naming the country where the study was conducted. Of these 31 selected articles, 16 frailty assessment instruments with relevant social indicators were reviewed. Eight frailty instruments were published/designed in Chinese, five published in Japanese, two in Korean, and one in English. Table 3 presents the summary of the included study. | Results; Page 8; Line 261-271. |
| Risk of bias in studies | 18 | Some of the reviewed screening scales from the Asia-Pacific settings did not disclose apparent validity and reliability checks. However, the authors used methodological rigor and only included quality studies by applying the quality assessment framework mentioned above. | Discussion; Page 13; Lines 243-237. |
| Results of individual studies | 19 | Individual studies data is presented in Supplementary Document 2. | Supplementary Document 2 |
| Results of syntheses | 20a | (1) Frailty assessment tools  This section describes the first theme regarding the assessment tools designed to measure social frailty syndrome in older adults in Asia-Pacific communities. The study reviewed sixteen frailty assessment tools with six social domains of frailty that assess social frailty syndrome in older people living in Asia-Pacific communities. Generally, the social domain of these assessment instruments showed heterogeneous characteristics regarding the number of items, which varied between 2 and 17. The most frequently utilized scale was SFQ by Makizako et al. (2015). This was reported in 13 studies, followed by SFQ by Teo et al. (2017) and SFQ for Yamada & Arai (2018) in three studies, while the other scales were observed in one study each. In addition, several studies reviewed used the SFQ for Makizako et al. (2015) where it demonstrated high efficiency in predicting adverse outcomes and mortality in community-living older adults (Bae et al., 2018; Hironaka et al., 2020; Tsutsumimoto et al., 2017; Yoo et al., 2019). However, Armstrong et al. (2015) showed that the SVI was the only assessment tool capable and likely to cover a broader range of social domains and items. Nine scales, including SFQs (n= 5 Social Frailty Questionnaire for Lian et al., 2020; Pek et al., 2020; Yamada & Arai, 2018; Teo et al., 2017; Makizako et al., 2015), Chinese LSNS-6, HALFT, FD-SF, and SVI, were 100% designed to measure the social frailty syndrome in older people.  (2) Social domains of frailty  The findings of this review revealed a great diversity of social items arranged in six main domains included in the available Asia-Pacific screening tools for assessing frailty syndrome in community-dwelling older adults. The social network was the most utilized domain in the reviewed scales (n=13 screening tools; n=29 study), followed by social activities (n=12 screening tools; n=28 study), social support (n=11 screening tools; n=13 analysis), financial difficulty (n=6 screening tools; n=10 studies), social role (n=4 screening tools; n=16 studies), and sociodemographic (n=2 screening tools; n=4 studies), such as education status and housing type. Therefore, social resources and social needs were the most frequent category in the reviewed tools (n= 13 tools each), followed by social behaviors (n= 12 tools) and general resources (n= 6 tools).  (3) Adverse outcomes related to social frailty and its domains  The study revealed eleven adverse health outcomes (n= 11 codes) associated with the social domains of frailty: mortality (n= 6 domains), physical frailty or mobility issues (n= 6 domains), disability incidence (n= 6 domains), cognitive frailty (n= 5 domains), mental illness or depression (n= 5 domains), nutritional disorders (n= 5 domains), dementia (n= 4 domains), oral frailty (n= 3 domains), hearing loss (n= 3 domains), BMI or obesity issues (n= 3 domains), and chronic pain (n= 3 domains). Thus, all six mentioned domains of social frailty predicted mortality, physical frailty, and disability incidence. | Results; Pages 8-10. |
| Reporting biases | 21 | Some of the reviewed screening scales from the Asia-Pacific setting lacked or did not disclose apparent validity and reliability checks. However, the authors used methodological rigor and only included quality studies by applying the quality assessment framework of Wells et al. (2000). | Discussion; Limitations and future research directions; Page 15. |
| Certainty of evidence | 22 | High, refer to the inclusion/exclusion criteria above. | NA |
| **DISCUSSION** | | |  |
| Discussion | 23a | The current systematic review proposed a model for characterizing social frailty domains and related adverse outcomes based on a holistic overview of 16 screening tools for frailty syndrome from Asian-Pasfic settings. It contributes to a comprehensive multi-dimensional definition of social frailty by providing a broad overview of the screening tools assessing social frailty in older people in the Asian-Pacific and by characterizing the related social domains and health outcomes. It explains social frailty as a lack of one or more aspects of social resources, social needs, social activities, and general needs, such as financial resources and socioeconomic difficulties, in older age, leading to several physical, mental, and cognitional health disorders and even mortality. | Discussion; Pages 11 & 12. |
|  | 23b | The limitations found in this review were the ambiguity between the adverse outcomes and the social domains, where some studies using the same frailty scale found a significant result about some of the possible outcomes, while others did not. One additional limitation identified in the study pertained to the potential confusion arising from the inconsistent nomenclature employed for specific scales across various research investigations. Moreover, many of the reviewed screening scales from the Asia-Pacific setting lacked or did not disclose clear validity and reliability checks. However, the authors used methodological rigor and only included quality studies by applying the quality assessment framework of Wells et al. (2000). Finally, all the studies reviewed were written in English and published within the last 20 years. Therefore, authors excluded from the review investigations that may have been reported in other (Asian) languages and published before 2002 were excluded. | Discussion: Limitations and future research directions; Page 15. |
|  | 23c | Based on these limitations, future research is recommended to develop a comprehensive empirical study to test the conceptual model of social frailty and its domains. A new comprehensive social assessment tool is required to contribute to the operationalization framework and a unified conceptual definition of social frailty. Future interventions that target individuals or groups of socially frail older adults should also address the several domains of social frailty mentioned in this study. Besides, future studies must apply a certain level of reliability and validity to the assessment tools. | Discussion: Limitations and future research directions; Page 15. |
|  | 23d | (a) Theoretical implication  The current systematic review, based on the existing evidence and theories, such as the Social Production Function theory and social frailty concept by Bunt et al. (2017), fills the gap in the literature by considering a wide range of social domains in the social frailty model and determining the related adverse health outcomes. The current systematic review contributes to the conceptual definition of social frailty and the interpretation and convergence of the concept of multidimensional social frailty. It also provides a better understanding of the role of each social domain in the conception of social frailty. Although the social variables of frailty, such as social activities and social resources, have been reviewed in the older people's frailty models (Bessa et al., 2018; Bunt et al., 2017), its related health outcomes are still unexplored, especially in the context of Asian-Pacific settings. Thus, the current review is one of the early studies that contributed to the concept of social frailty, its domains, and its health outcomes in Asia-Pacific communities. It also explored the significance of including the different social domains reviewed in defining the state of frailty and the related adverse health outcomes. Therefore, it contributes to the theoretical model of social frailty proposed by previous evidence by reviewing the screening tools that contributed to characterizing the social domains and identifying their related adverse health outcomes. Besides, the significance of this review lies in linking social health science to public health and health sciences.  (b) Practical implication  The current study proved that each domain of the six reviewed domains of social frailty predicts adverse health outcomes, including mortality, especially in the Asian-Pacific. It characterized the different health outcomes based on each domain of social frailty and linked them to specific screening tools. It enhanced understanding of the common negative health outcomes resulting from the frailty of each social domain. This facilitates professionals and researchers in making informed decisions regarding selecting instruments tailored to each study's specific circumstances and scope. This also contributes to guiding them to develop therapies designed to mitigate or postpone the onset of social frailty. Therefore, professionals and researchers in health and social sciences are encouraged to consider the six mentioned domains to understand better the frailty syndrome and related health consequences. Furthermore, the current review provided a theoretical background for professionals and researchers to understand social frailty syndrome in older people. | Discussion: Theoretical implication & Practical impication; Page 14-15; Lines 472-497. |
| **OTHER INFORMATION** | | |  |
| Registration and protocol | 24 | PROSPERO was searched to ensure a similar systematic review study protocol was not registered. No prior studies focusing on the current topic of interest were identified. Thus, the current systematic search protocol was registered with PROSPERO (the registration number is CR42021225980). | Literature Survey Methodology; Page 5; Lines 151-152. |
| Support | 25 | This study was part of the Transforming Cognitive Frailty to Later-Life Self-sufficiency (AGELESS) study funded by the Ministry of Higher Education Malaysia Long-Term Research Grant Scheme (LRGS/1/2019/UM/01/1/2), which has evolved from the LRGS TUA Study. The study was also supported by the Research Management Centre (RMC), Universiti Putra Malaysia (UPM). | - |
| Competing interests | 26 | The authors declare that they have no competing interests. | - |
| Availability of data, code and other materials | 27 | Supplementary documents have been attached. | Supplementary documents 1, 2, 3,& 4. |

*From:*  Page MJ, McKenzie JE, Bossuyt PM, Boutron I, Hoffmann TC, Mulrow CD, et al. The PRISMA 2020 statement: an updated guideline for reporting systematic reviews. BMJ 2021;372:n71. doi: 10.1136/bmj.n71
