# Supplementary material for: Characterization of social frailty domains and related adverse health outcomes in the Asia-Pacific: a systematic literature review
Source: PeerJ. 2024 Mar 15;12:e17058. doi: 10.7717/peerj.17058 (PMC10946386; doi:10.7717/peerj.17058)
Supplement: Supplemental Information 2 [file peerj-12-17058-s002.docx]

**Supplementary Documents 2:** Summary on the selected studies.

| Source | Study objective | Method | Screening tool | Variable | Finding |
| --- | --- | --- | --- | --- | --- |
| Armstrong et al. (2015) | To evaluate the mortality risk related to social frailty among older Japanese men. | ***Design:*** a cross-sectional study (Honolulu-Asia Aging Study)  ***Year:*** 1995  ***Sample:*** n= 3,271; age: ≥72  ***Setting:*** Japan | **1. Social Vulnerability Index (SVI 17-item) for social frailty**  2. Frailty Index (FI 58-variables)  3. Mortality risk: date of death collected up to 21 years from baseline | ***DV:*** Mortality risk  ***IV:*** Social vulnerability/frailty  ***Covariate:*** Demographics and lifestyle factors | Social vulnerability was associated with mortality risk. Besides, each social deficiency was associated with a 5% increase in mortality risk in frail older adults. |
| Kawn et al. (2015) | To evaluate the validity of Comprehensive Model of Frailty (CMF) in predicting functional dependency and self-rated health. | ***Design:*** a cross-sectional study (Hong Kong Centenarian Study)  ***Year:*** 2011  ***Sample:*** n= 124; age: ≥60  ***Setting:*** Hong Kong | **1. Comprehensive Model of Frailty (CMF)**  2. Self-rated health scale  3. Activities of daily living and dependency | ***DV:*** Self-rated health  ***IV:*** Frailty  ***Covariate:*** Demographics | The presence of social frailty was associated with a wide range of lower self-rated health, including mortality. |
| Makizako et al. (2015) | To examine the relationship between disability onset and social frailty among community older people. | ***Design:*** a longitudinal study (Obu Study of Health Promotion for the Elderly)  ***Year:*** 2011-2012  ***Sample:*** n= 4304; age: ≥65  ***Setting:*** Japan | **1. 5-item Social Frailty Scale developed by the same author**  2. Onset of disability: LTCI system that categorizes people into 7 levels of needs | ***DV:*** Onset of disability based on 7- levels of needs  ***IV:*** 5-item of Social Frailty  ***Covariate:*** Demographics and medical history | Social frailty based on five social items, including living alone, visiting friends often, going out less than in the prior year, talking with someone daily, and feeling helpful to others significantly affected the development of disability in older adults. |
| Ma et al. (2017) | To investigate the association among frailty risk factors in Chinese older adults. | ***Design:*** a cross-sectional (China Comprehensive Geriatric Assessment Study)  ***Year:*** 2011-2012  ***Sample:*** n= 5,844; age: ≥60  ***Setting:*** 7 Cities in China | **1. The Comprehensive Geriatric Assessment-Frailty Index (CGA-FI; 5-domains)**  2. Chronic disease and geriatric syndromes  3.Physical activities | ***DV:*** Morbidity, multimorbidity  ***IV:*** Frailty  ***Covariate:*** Demographics, lifestyle, and medical factors | ADL impairment was the strongest risk factor for physical frailty variable, after controlling the covariates. Social frailty risk was also associated with several risks, such as depression, mobility, and disability incidence. |
| Teo et al. (2017) | To evaluate the relationship between the social frailty phenotype and functional disability and compare the abilities of the social frailty and physical frailty in predicting functional disability. | ***Design:*** a longitudinal study (Singapore Longitudinal Ageing Studies)  ***Year:*** 1^st^ wave (2003-2005)  2^nd^ wave (2009-2009)  ***Sample:*** n= 2,406  ***Setting:*** Singapore | **1. 8-item Social Frailty Scale developed by the same author**  2. , | ***DV:*** Functional disability  ***IV:*** Physical frailty and social frailty  ***Covariate:*** Demographics, medical history, lifestyle, and nutritional risk | Social frailty was significantly associated with a significant increase in disability and severe disability than older people without social frailty. |
| Tsutsumimoto et al. (2017) | To determine the association between social frailty, physical and cognitive function among community-dwelling older adults. | ***Design:*** a cross-sectional study  ***Year:*** 2011  ***Sample:*** n= 4425; age: ≥65  ***Setting:*** Obu City, Japan | **1. 5-item Social Frailty Scale by Makizako et al. (2015)**  2. NCGG-FAT to measure 4-domains of cognitive function  3. Walking speed and grip strength scale | ***DV:*** Social Frailty  ***IV:*** Cognitive function and physical function  ***Covariate:*** Demographics, medical histories, lifestyle, and depressive symptoms | Social frailty was significantly negatively associated with all cognitive function tests and physical function deficits among older Japanese people. |
| Bae et al. (2018) | To investigate the role of social frailty in the association between mild cognitive impairment and hearing problems. | ***Design:*** a cross-sectional study  ***Year:*** 2015  ***Sample:*** n= 4251; age: ≥65  ***Setting:*** Obu City, Japan | **1. Social Frailty (SF 5- item of Makizako et al. (2015)**  2. Cognitive function using 4-domains of NCGG-FAT  3. Hearing problems using 10- item Hearing Handicap Inventory for the Elderly and for Adults - Screening version (HHIE-S) | ***DV:*** Cognitive function  ***IV:*** 5- item of Social Frailty and hearing problems  ***Covariates:*** Demographics, medical histories, and lifestyle | Social frailty was correlated with multiple factors of mild cognitive impairment (MCI). The co-occurrence of hearing difficulties and social frailty was strongly linked to the highest risk of multi-domain MCI. |
| Ma et al. (2018) | To evaluate the relationship between social frailty and physical functioning, depression, cognition, and mortality among community older adults. | ***Design:*** a longitudinal study (Beijing Longitudinal Study of Aging)  ***Year:*** 1^st^ wave (2004)  2^nd^ wave (2012)  ***Sample:*** n= 1,697; age: ≥60  ***Setting:*** Beijing, China | **1. Social Frailty Screening a 5-item Questionnaire of HALFT scale**  2. Physical frailty: assessed using a frailty index of 68-parameters  3. Dementia: using a self-reported history diagnosed by a doctor  4. Cognition: MMSE  5. Depression: CES-D | ***DV:*** Physical function, dementia, and depression  ***IV:*** 5-item of HALFT  ***Covariate:*** Demographics and lifestyle, and satisfaction | Social frailty was significantly correlated with cognition deficits and dementia, depression, oral frailty, physical function, and predicted mortality. The HALFT provided a useful screening scale for determining social frailty in older people. |
| Makizako et al., (2018a) | To investigate the relationship between social frailty and loss of muscle mass and weakness among community older adults. | ***Design:*** cross-sectional study  ***Year:*** 2018  ***Sample:*** n= 353; age: ≥65  ***Setting:*** Tarumizu City, Japan | **1. Social Frailty (SF 5-item) a self-reported questionnaire**  2. Muscle & weakness: multi-frequency bioelectrical impedance analysis & Smedleytype handheld dynamometer | ***DV:*** 5- item of Social Frailty  ***IV:*** Muscle mass and dominant handgrip strength  ***Covariate:*** Demographics and medical history | Social frailty was correlated with a prevalence of muscle weakness and loss of skeletal muscle mass. However, it was unrelated to skeletal muscle mass function after controlling covariate variables. |
| Makizako et al. (2018b) | To investigate the impact of social frailty on the development of physical frailty among older adults. | ***Design:*** a longitudinal study (Obu Health Promotion for the Elderly Study)  ***Year:*** 1^st^ wave (2011-2012)  2^nd^ wave (2015-2019)  ***Sample:*** n= 1226; age: ≥65  ***Setting:*** Obu City, Japan | **1. Social Frailty (SF 5-item) a self-reported questionnaire**  2. Physical Frailty Phenotype (5-conditions: slowness, weakness, exhaustion, levels of activity, and weight loss | ***DV:*** 5- item of Social Frailty  ***IV:*** 5-condition of Physical Frailty  ***Covariate:*** Demographics and medical histories | Social frailty was significantly related to walking speeds, contributing to physical frailty in a relatively short time among older people who are not physically frail. Social frailty can also be related to age-related changes in movement and mobility. |
| Qiao et al. (2018) | To develop a cross-culturally adapt the Comprehensive Frailty Assessment Instrument (CFAI) among the Chinese community. | ***Design:*** a cross-sectional study  ***Year:*** 2015  ***Sample:*** n= 1,235; age: ≥60  ***Setting:*** Jinan City, China | **Chinese Comprehensive Frailty Assessment Instrument (CFAI, 4-components) to assess frailty** | ***V1:*** Four components frailty  ***V2:*** Participants’ socio-demographic characteristic: age, gender, education, marital status, income, and living arrangement | The Chinese CFAI provides good validity and reliability as a practical frailty scale that can be used easily to measure various parameters of frailty and health outcomes among community-dwelling older people. |
| Tsutsumimoto et al. (2018) | To evaluate the association between the incidence of depressive symptoms and types of frailty among community older people. | ***Design:*** a longitudinal study (National Center for Geriatrics and Gerontology)  ***Year:*** 1^st^ wave (2011-2012)  2^nd^ wave (2015-2016)  ***Sample:*** n= 3,538; age: ≥65  ***Setting:*** Obu City, Japan | **1. Social Frailty (SF 5-item of Makizako et al. (2015)**  2. Cognitive function using 4-domains of NCGG-FAT: memory, attention, executive function, and processing speed  3. Physical function: measuring walking speed and grip strength  4. Depression: GDS Checklist | ***DV:*** Depressive symptoms  ***IV:*** Social Frailty, Cognitive function and physical function  ***Covariates:*** Demographics and medical history | The multivariable regression analysis proved that symptoms of depression were significantly correlated with social frailty but not physical or cognitive frailty. |
| Yamada & Arai (2018) | To identify the prevalence of social frailty and its correlation to disability and mortality in community Japanese older people. | ***Design:*** a longitudinal study (primary data)  ***Year:*** 1^st^ wave (2011)  2^nd^ wave (2017)  ***Sample:*** n= 6,603; age: ≥65  ***Setting:*** Shiga City, Japan | **1. Social Frailty (SF 4- item) a self-reported questionnaire**  2. A simple frailty screening index (5-item) to assess physical/psychological frailty  3. Onset of disability using LTCI  4. Death using LTCI | ***DV:*** 4-item of social frailty  ***IV:*** 5-item of physical /psychological frailty  ***Covariate:*** Demographics and medical history | Social frailty was significantly associated with several health outcomes; it was also significantly associated with high risk for incident disability and mortality. |
| Abe et al. (2019) | To examine the frailty in different areas and associated factors. | ***Design:*** a cross-sectional study (baseline survey)  ***Year:*** 2016-2018  ***Sample:*** n= 9182; age: ≥65  ***Setting:*** metropolitan, suburban, and rural areas of Japan | **1. Frailty: Kaigo-Yobo Checklist (KYCL) 15-item**  2. Association factors measurement | ***DV:*** three domains of frailty  ***IV:*** a) Association factors: age, weight, medical history, living arrangement, and economic status. b) Location of participants | The social frailty was significantly related to lower nutrition, physical activity, economic status, bone and joint disease, and mortality. People with high levels of frailty also had lower social activities and malnutrition. |
| Hirase et al. (2019) | To evaluate the association between chronic pain and social frailty among community older adults. | ***Design:*** a cross-sectional study  ***Year:*** 2018-2019  ***Sample:*** n= 248; age: ≥65  ***Setting:*** Japan | **1. Social Frailty (SF 5-item of Makizako et al. (2015)**  2. Chronic pain using documenting pain sites and intensity at the site of maximum pain  3. Physical function was assessed using the CST | ***DV:*** Chronic pain  ***IV:*** 5-item of Social Frailty  ***Covariate:*** Demographics and physical function | Social frailty was significantly associated with chronic pain, especially poor CST performance. Chronic pain was mainly related to three social domains: going out less frequently, feeling unhelpful to friends or family, and rarely visiting friends. |
| Kuo et al. (2019) | To develop a social frailty prediction machine system to identify social frailty among elders. | ***Design:*** a cross-sectional study  ***Year:*** 2017  ***Sample:*** n= 595; age: ≥65  ***Setting:*** Kaohsiung City, Taiwan | **1. Social Frailty using 6-item of Chinese LSNS-6**  2. Depression using the Geriatric Depression Scale (GDS-5) | ***V1:*** 6-item social frailty  *V2:* Depression, medical histories, physical activity, and protein intake | The health literacy variable was the most important for predicting social frailty, followed by comorbidity and religious participation, physical frailty, and geriatric depression, respectively. |
| Nakakubo et al. (2019) | To examine the association between social frailty and sleep duration and excessive daytime sleepiness. | ***Design:*** a longitudinal study (National Center for Geriatrics and Gerontology – Study of Geriatric Syndromes)  ***Year:*** 2011-2012  ***Sample:*** n= 4,427; age: ≥70  ***Setting:*** Obu City, Japan | **1. Social Frailty (SF 5-item of Makizako et al. (2015)**  2. Self-reported sleep duration to assess sleep duration and Ehlers-Danlos syndrome  3. Depression using the Geriatric Depression Scale (GDS) | ***DV:*** 5-item Social Frailty  ***IV:*** Sleep duration and Ehlers-Danlos syndrome  ***Covariate:*** Demographics and medical history | The severity of long sleep duration was significantly associated with the possibility of social frailty. Long sleep duration could be as a result of physical or cognitive frailty. |
| Park et al. (2019) | To assess the association between social frailty and physical frailty, geriatric syndromes, and ADL disability in community older people. | ***Design:*** a cross-sectional (The Aging Study of Pyeong-Chang Rural Area)  ***Year:*** 2018  ***Sample:*** n= 408; age: ≥65  ***Setting:*** rural area in South Korea | **1. Social Frailty (SF 5-item of Makizako et al. (2015)**  2. Cardiovascular Health Study (CHS) scale used to measure physical frailty  3. Geriatric conditions: various scales: Malnutrition using MNA-SF; Cognitive using MMSE; Depression using CESD; Physical function using gait speed. | ***DV:*** Geriatric conditions  ***IV:*** 5-item Social Frailty  ***Covariate:*** Demographics, living condition, and lifestyle | Social frailty was significantly associated with the increased risk of disability, depression, and physical frailty. Social frailty was also correlated with living alone, age, lack of accessibility to facilities and neighbours, and low-income levels. |
| Teo et al. (2019) | To evaluate the hypothesis, “the addition of mental frailty and social frailty to physical frailty increases the ability to predict various health outcome.” | ***Design:*** a longitudinal study (Singapore Longitudinal Ageing Studies)  ***Year:*** 2003 and 2007  ***Sample:*** n= 2,387  ***Setting:*** Singapore | **1. Social Frailty (SF 8-item) a self-reported questionnaire**  2. Physical frailty (PF) using Fried’s criteria  3. Mental frailty (MF): 3-domains (cognitive; mood; self-rated health)  4. Nutrition using Nutrition Screening Initiative (NSI)  5. Mortality using observation the mortality date.  6. Adverse health outcomes scale | ***DV:*** Adverse health outcomes  ***IV:*** Social frailty, physical frailty, and mental frailty  ***Covariate:*** Demographics, medical morbidity, lifestyle, and nutritional risk | Each frailty, including social frailty, was independently correlated with the prevalence of functional and severe disability, oral and physical defects, and mortality. Adding social and mental frailties to physical fragility increased risk estimates by more than twofold. |
| Tsutsumimoto et al. (2019) | To assess the relationship between social frailty and Alzheimer’s disease (AD) among community older adults. | ***Design:*** a longitudinal study  ***Year:*** 1^st^ wave (2011-2012)  2^nd^ wave (2015-2016)  ***Sample:*** n= 3,720; age: ≥65  ***Setting:*** Obu City, Japan | 1. Social frailty (SF 5-item of Makizako et al. (2015)  2. Alzheimer’s disease: Alzheimer’s disease diagnoses during the 53-month follow-up  3. Dementia status: based on medical diagnosis from a medical doctor | ***DV:*** Alzheimer and dementia  ***IV:*** 5-item of Social Frailty  ***Covariate:*** Demographics and medical histories | Social frailty was significantly correlated with a higher cumulative Alzheimer’s disease and dementia incidence risk in older Japanese people. |
| Yoo et al. (2019) | To evaluate the association between hearing loss and social frailty in community older adults. | ***Design:*** a cross-sectional (Korean Frailty and Aging Cohort Study)  ***Year:*** 2016-2018  ***Sample:*** n= 3,014; age: ≥70  ***Setting:*** South Korea | **1. Social Frailty (SF 5-item of Makizako et al. (2015)**  2. The pure-tone audiometry was performed in a booth using various frequencies of Hz  3. Physical frailty (PF) using 5-item of Fried’s phenotype | ***DV:*** 5-item of Social Frailty  ***IV:*** Hearing loss  ***Covariate:*** Demographics, medical history, and physical frailty | Domains of social frailty, including social activities and social support, were significantly related to the risk of hearing loss. The odds ratio of social defects to hearing loss was high after adjusting physical frailty and confounding variables. |
| Hironaka et al. (2020) | To investigate the relationships between oral frailty, physical frailty, and social frailty in community-dwelling older people. | ***Design:*** a longitudinal study (Tokyo Metropolitan Institute of Gerontology)  ***Year:*** 2014-2017  ***Sample:*** n= 666; age: ≥60  ***Setting:*** Nagoya City, Japan | **1. Social Frailty (SF 5-item of Makizako et al. (2015)**  2. Physical frailty: 5-conditions: slowness, weakness, exhaustion, low levels of activity, and weight loss  3. Oral frailty: 4-item: number of teeth present; masticatory performance; tongue pressure; and oral diadochokinesis (ODK)  4. Physical frailty (PF) using Fried’s criteria  5. Cognitive using MMSE | ***DV:*** Oral Frailty  ***IV:*** Social and Physical frailty  ***Covariate:*** Demographics, nutritional status, cognitive psychological function, and medical history | Social frailty was significantly related to oral frailty, and both social and oral frailties were significantly associated with physical frailty. Social frailty was also significantly related to the Mini Nutritional Assessment, age, and polypharmacy. |
| Huang et al. (2020) | To evaluate the correlation between social frailty and diet quantity, diet quality, and nutrition over a three-year period in Japanese older adults. | ***Design:*** a cross-sectional (Otasha Kenshin)  ***Year:*** 2011  ***Sample:*** n= 682; age: ≥65  ***Setting:*** Tokyo City, Japan | **1. Social Frailty (SF 4-item of Yamada & Arai (2018)**  2. Nutritional status: Mini-Nutritional Assessment (MNA) tool  3. Diet quality and quantity: 34-item of food frequency questionnaire | ***DV:*** Nutritional status  ***IV:*** 4-item of social frailty  ***Covariate:*** Demographics, physical function, and medical history | Overall, social frailty was correlated poor nutrition in Japanese community-dwelling older men. This could be also related to oral problems. |
| Lee et al. (2020) | To determine the of social deficits and their combined effects with social frailty in predicting functional decline and mortality in community older people. | ***Design:*** a longitudinal study (Living Profiles of Older People Survey)  ***Year:*** 1^st^ wave (2008)  2^nd^ wave (2011)  ***Sample:*** n= 11,241; age: ≥65  ***Setting:*** South Korea | **1. Social Deficits (SD-SF 9-item) a self-reported questionnaire**  2. Physical Frailty using Fried Phenotype Model  3. Dementia: a self-reported history of dementia diagnosed by a doctor | ***DV:*** 5-item of physical frailty  ***IV:*** 9-item of social deficits  ***Covariate:*** Demographics, health, and chronic conditions | Social deficits increase the risk of adverse outcomes associated with physical frailty and elevated mortality. Combining social deficits and physical frailty increases hazards and the highest mortality risk. |
| Lian et al. (2020) | To examine the association between frailty markers and socioeconomic status to indicate depressive symptoms. | ***Design:*** a longitudinal study (nationally representative longitudinal survey of Chinese community)  ***Year:*** 1^st^ wave (2011)  2^nd^ wave (2013)  3^rd^ wave (2015)  ***Sample:*** n= 6,641; age: ≥60  **Setting:** China | **1. Social frailty (SF 2-item) self-reported questionnaires**  2. Physical frailty: based on the Cardiovascular Health Study measuring weakness and slowness  3. Depression using Center for Epidemiologic Studies Depression Scale (CESD) | ***DV:*** Depression  ***IV:*** Socioeconomic status  ***MV:*** Social & Physical frailty  ***Covariate:*** Demographics and medical histories | Socioeconomic status was associated with depression through social frailty and physical frailty sequentially. Social activities and social frailty were related to the incidence of depressive symptoms, especially in higher education levels and non-agricultural work. |
| Nagai et al. (2020) | To elucidate the effect of physical frailty on social frailty and identify the physical frailty domain that predicts social frailty. | ***Design:*** a longitudinal study of the Frail Elderly in the Sasayama-Tamba Area  ***Year:*** 1^st^ wave (2015-2017)  2^nd^ wave (2017-2019)  ***Sample:*** n= 342; age: ≥65  ***Setting:*** Tamba-Sasayama, Japan | **1. Social frailty (SF 4-item of Yamada & Arai (2018)**  2. Physical frailty (PF) using Fried’s phenotype: slowness, weakness, exhaustion, low activity, and weight loss  3. Depression using Geriatric Depression Scale (GDS) | ***DV:*** 4-item of social frailty  ***IV:*** 5-item of physical/psychological frailty  ***Covariate:*** Demographics and medical histories | In general, physical frailty, including gait speed and weakness, predicted the development of social defects in older people. Physical variables were independent risk factors associated with developing a future decline in social frailty. |
| Pek et al. (2020) | To evaluate the relationship between social frailty and outcomes of mood, nutrition, physical activity, physical performance, and life-space mobility. | ***Design:*** a cross-sectional study (secondary data of GeriLABS 2)  ***Year:*** 2017-2019  ***Sample:*** n= 229; age: ≥60  ***Setting:*** Asian community-dwelling older people | **1. Social Frailty (SF 9-item) a self-reported questionnaire**  2. Geriatric Depression Scale (GDS)  3. Mini Nutritional Assessment  4. Physical Performance Battery  5. International Physical Activity  6. Life–Space Assessment  7. Physical frailty: Fried’s phenotype | ***DV:*** Mood; nutrition; physical performance; physical activities; life-space mobility  ***IV:*** 9-item of social frailty  ***Covariate:*** Demographics and medical factors | Social pre-frailty was associated with poor physical function and low physical activity. Social frailty was related to malnutrition risk, depression symptoms, low physical activity, and poor physical performance. Social frailty also was associated with various adverse health outcomes. |
| Song et al. (2020) | To evaluate the correlation between multidimensional frailty and hypertension and between frailty and obesity in older people living in community. | ***Design:*** a cross-sectional study (secondary data)  ***Year:*** 2016-2017  ***Sample:*** n= 995; age: ≥65  ***Setting:*** Zhengzhou City, China | **1. Tilburg Frailty Indicator (4 domains) to assess frailty**  2. BMI and waist circumference to assess lifestyle | ***DV:*** Lifestyle  *IV:* Four domains of frailty  ***Covariate:*** Demographics and medical status | The multidimensional frailty was correlated to older adults’ hypertension. Social frailty, multidimensional frailty and psychological frailty were positively associated with general obesity and abdominal obesity. |
| Watanabe et al. (2020) | To examine the association between the prevalence of frailty and BMI in Japanese older people. | ***Design:*** a cross-sectional study (Kyoto–Kameoka Study)  ***Year:*** 2011-2012  ***Sample:*** n= 7191; age: ≥65  ***Setting:*** Kameoka City, Japan | **1. Frailty: Kihon Checklist (KCL) 25-item & Frailty Phenotype 5-item**  2. BMI: self-reported body weight (kg) by the square of height (m) | ***DV:*** Frailty  ***IV:*** BMI  ***Covariate:*** Demographics, lifestyle, and medical history | Frailty, including social frailty, was significantly associated with the BMI range. A healthy BMI can contribute to reducing the frailty prevalence, including the social frailty. |
| Kim et al. (2021) | To investigate the experts' opinion in the development of older adults' frailty assessment scale. | ***Design:*** qualitative interviews  ***Year:*** 2016-2017  ***Sample:*** n= 46 experts  ***Setting:*** South Korea | **Frailty assessment scale (FAS, 9-item) a self-reported questionnaire** | NA | Overall, there is an expert consensus on the included items of a frailty assessment. In addition, a consensus was reached on the need for a broad phenotype, including physical, social, and mental frailties. |
| Miyata et al. (2022) | To investigate the associations between satisfaction with older adults’ activities and social frailty. | ***Design:*** a cross-sectional study (Tarumizu Study 2019)  ***Year:*** 2019  ***Sample:*** n= 596; age: ≥65  ***Setting:*** Tarumizu City, Japan | **1. Social Frailty: SF 5-item of Makizako et al. (2015)**  2. Aid for Decision-Making in Occupational Choice (ADOC) measuring 95 activities. | ***DV:*** 5-item of social frailty  ***IV:*** 95 meaningful activities in older age | Overall, social frailty significantly associated with lower satisfaction with meaningful activity, depressive symptoms, and poor higher-level competence. |
| DV: Dependent variable, IV: Independent variable, MV: Moderating variable, V1: Variable 1, V2: Variable 2, LTCI: Long-term care insurance, IADL: Instrumental Activities of Daily Living, ADL: Activities of Daily Living, NCGG-FAT: National Center for Geriatrics and Gerontology-Functional Assessment Tool, HALFT: an acronym for the five components “Help”, “Participation”, “Loneliness”, “Financial” and “Talk”, CST: Chair Stand Test, LSNS-6: Lubben Social Network Scale, GeriLABS 2: Longitudinal Assessment of Biomarkers for characterization of early Sarcopenia and Osteosarcopenic Obesity in predicting frailty and functional decline in community-dwelling Asian older adults Study. | | | | | |
