# Supplementary material for: Characterization of social frailty domains and related adverse health outcomes in the Asia-Pacific: a systematic literature review
Source: PeerJ. 2024 Mar 15;12:e17058. doi: 10.7717/peerj.17058 (PMC10946386; doi:10.7717/peerj.17058)
Supplement: Supplemental Information 3 [file peerj-12-17058-s003.docx]

**Supplementary Document 3:** Quality assessment using the Newcastle-Ottawa Scale (NOS)

| **Study** | **Selection** |  |  |  | **Comparability** | **Outcome** |  | **Score** |
| --- | --- | --- | --- | --- | --- | --- | --- | --- |
|  | Representativeness of the sample | Sample size justified | on-respondents | Ascertainment of exposure (max**) | Confounding controlled (max**) | Outcome assessment (max**) | Statistics | **Total** |
| Study 1 |  | * | * | ** | ** | ** | * | 9 |
| Study 2 | * |  | * | * | ** | ** | * | 9 |
| Study 3 | * |  | * | ** | * | ** | * | 8 |
| Study 4 | * | * | * | * | * | ** | * | **9** |
| Study 5 | * |  | * | ** | ** | ** | * | 9 |
| Study 6 | * |  | * | ** | ** | ** | * | 10 |
| Study 7 | * | * | * | ** | ** | ** | * | 10 |
| Study 8 | * | * | * | ** | ** | ** | * | 10 |
| Study 9 | * | * | * | ** | * | ** | * | 9 |
| Study 10 | * | * | * | ** | ** | ** | * | 10 |
| Study 11 | * | * | * | ** | ** | ** | * | 10 |
| Study 12 | * |  | * | ** | * | ** | * | 8 |
| Study 13 | * |  | * | ** | ** | ** | * | 9 |
| Study 14 | * | * | * | * | * | ** | * | 9 |
| Study 15 | * | * | * | ** | ** | ** | * | 10 |
| Study 16 | * |  | * | ** | ** | ** | * | 9 |
| Study 17 | * | * | * | * | ** | ** | * | 9 |
| Study 18 | * | * | * | ** | * | ** | * | 9 |
| Study 19 | * |  |  | * | * | * | * | 5 |
| Study 20 | * |  | * | ** | * | ** | * | 8 |
| Study 21 | * | * | * | ** | ** | ** | * | 10 |
| Study 22 | * | * | * | ** | ** | ** | * | 10 |
| Study 23 | * |  | * | * | * | ** | * | 7 |
| Study 24 | * |  | * | ** | ** | ** | * | 9 |
| Study 25 | * |  |  | * | * | * | * | 5 |
| Study 29 | * | * | * | ** | ** | ** | * | 10 |
| Study 30 | * | * | * | ** | ** | ** | * | 10 |
| Study 31 | * |  |  | * | * | ** | * | 6 |
| Study 32 | * |  | * | ** | * | ** | * | 8 |
| Study 33 | * | * | * | ** | ** | ** | * | 10 |
| Study 34 | * | * | * | * | * | * | * | 7 |
| *High quality= 7-10; Moderate quality= 4-6; Low Quality= 0-3. The study should score at least “7” to be included in the review.* | | | | | | | | |
