# Supplementary figures and images for: Characterization of social frailty domains and related adverse health outcomes in the Asia-Pacific: a systematic literature review
Source: PeerJ. 2024 Mar 15;12:e17058. doi: 10.7717/peerj.17058 (PMC10946386; doi:10.7717/peerj.17058)

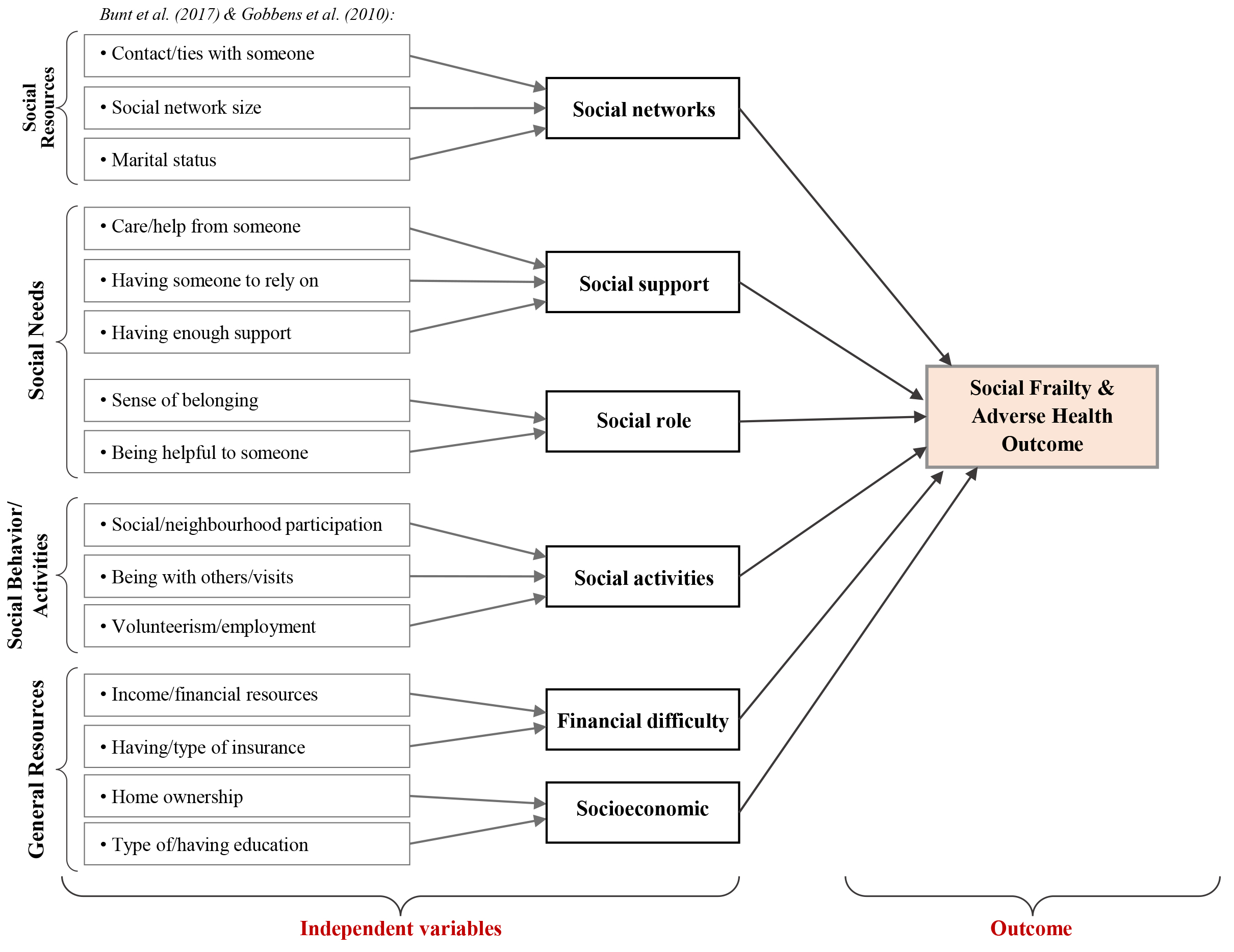

Supplement: Supplemental Information 4 [file peerj-12-17058-s004.jpg]
